# Spleen Tyrosine Kinase (Syk) Regulates Systemic Lupus Erythematosus (SLE) T Cell Signaling

**DOI:** 10.1371/journal.pone.0074550

**Published:** 2013-08-27

**Authors:** Alexandros P. Grammatikos, Debjani Ghosh, Amy Devlin, Vasileios C. Kyttaris, George C. Tsokos

**Affiliations:** 1 Division of Rheumatology, Beth Israel Deaconess Medical Center, Harvard Medical School, Boston, Massachusetts, United States of America; 2 Department of Microbiology and Immunology, the Brody School of Medicine, East Carolina University, Greenville, North Carolina, United States of America; University of Patras Medical School, Greece

## Abstract

Engagement of the CD3/T cell receptor complex in systemic lupus erythematosus (SLE) T cells involves Syk rather than the zeta-associated protein. Because Syk is being considered as a therapeutic target we asked whether Syk is central to the multiple aberrantly modulated molecules in SLE T cells. Using a gene expression array, we demonstrate that forced expression of Syk in normal T cells reproduces most of the aberrantly expressed molecules whereas silencing of Syk in SLE T cells normalizes the expression of most abnormally expressed molecules. Protein along with gene expression modulation for select molecules was confirmed. Specifically, levels of cytokine IL-21, cell surface receptor CD44, and intracellular molecules PP2A and OAS2 increased following Syk overexpression in normal T cells and decreased after Syk silencing in SLE T cells. Our results demonstrate that levels of Syk affect the expression of a number of enzymes, cytokines and receptors that play a key role in the development of disease pathogenesis in SLE and provide support for therapeutic targeting in SLE patients.

## Introduction

Following recognition of an antigen on the surface of a major histocompatibility complex (MHC) molecule, the T cell receptor (TCR) initiates a number of signaling cascades that determine cytokine production, cell survival, proliferation and differentiation. The initial event, phosphorylation of immunoreceptor tyrosine-based activation motifs (ITAMs) on the cytosolic side of the TCR/CD3ζ chain complex, allows for Zap70 (ζ-chain associated protein kinase) to be recruited to CD3ζ. Zap70 becomes activated in this way and promotes the recruitment and phosphorylation of other adaptor molecules responsible of transmitting signals downstream.

Several studies have shown that TCR signaling is modified in patients suffering from SLE [1,2]. Instead of transmitting signals through TCR to CD3ζ and Zap70, an alternative pathway comes into play involving FcRγ and spleen tyrosine kinase (Syk) [3,4]. FcRγ is homologous in shape and function to CD3ζ and takes its place in SLE T cells [5,6] and associates with Syk. This alternative FcRγ/Syk duet is 100 times enzymatically more potent than the canonical CD3ζ/Zap70. As a result, following activation, SLE T cells exhibit higher intracytoplasmic calcium flux and cytosolic protein tyrosine phosphorylation [7,8].

To better understand the contribution of Syk in the aberrant phenotype of SLE T cells we examined the effect of Syk on the expression of molecules known to contribute to the pathogenesis of SLE. A two-step approach was followed: (a) Syk was overexpressed in healthy blood-donor T cells to examine whether increased Syk expression creates SLE-like phenotype; and (b) Syk was downregulated, using siRNA, in SLE T cells to examine whether gene expression abnormalities can be corrected. Our results show that Syk contributes significantly to the abnormal expression of a number of molecules associated with the immunopathogenesis of SLE.

## Materials and Methods

### Ethics statement and blood samples

This study was approved by the Institutional Review Board of Beth Israel Deaconess Medical Center (BIDMC). Written informed consent was obtained from all participating subjects and all clinical investigation was conducted according to the principles expressed in the Declaration of Helsinki. Blood samples were obtained from 21 SLE patients attending the Rheumatology Division of BIDMC and 14 healthy blood donors from the Dana-Farber Cancer Institute. All participating patients fulfilled at least 4 out of 11 criteria for SLE as set forth by the American College of Rheumatology [9]. Patient characteristics are shown in Table 1. In each experiment samples from different patient or healthy control blood donors were used. The disease activity of the patients was determined using the Systemic Lupus Erythematosus Activity Index (SLEDAI) [10].

**Table 1:** Patient characteristics.

**SLEDAI**	mean ±SEM: 2 ±1 (range: 0-9)
**Sex**	95% female
**Race**	50% black; 35% white; 10% mixed; 5% asian
**Age**	mean ±SEM: 47 ±2 (range: 32-60)

### Cells, reagents and antibodies

Total T cells were purified using the Rosette Sep T cell kit (StemCell Technologies, Vancouver, Canada). Blood was incubated with a purification mixture that contains antibodies against CD14, CD16, CD19, CD56 and glyA and attaches non-T cells to erythrocytes. Lymphocyte separation medium (Cellgro, Manassas, VA) was subsequently used to separate these complexes from T cells.

For flow cytometry the following antibodies were used: SYK-PE from Santa Cruz Biotechnology (Santa Cruz, CA), CD3-PB from Biolegend (San Diego, CA), CD44v3-APC from R&D systems (Minneapolis, MN), CD44v6-FITC from Abcam (Cambridge, MA) and IL-21-AlexaFluor647 from BD Pharmingen (San Jose, CA).

For western blot the following antibodies were used: OAS2 from Proteintech (Chicago, IL), PP2A C subunit from Cell Signaling (Boston, MA), β-actin from Sigma-Aldrich (St. Louis, MO) and anti-rabbit HRP-conjugated secondary antibody from Santa Cruz Biotechnology (Santa Cruz, CA).

### Plasmid and siRNA transfections

Transient transfections of human T cells were carried out using the Lonza Nucleofector system (Lonza, Cologne, Germany). Briefly, 5 × 10^6^ cells were resuspended in 100µl of nucleofector solution, plasmid DNA (1µg/10^6^ cells) was added, and cells were transferred to cuvettes to be transfected using the U-014 program. The PCMV6XL6 -SYK expression plasmid from OriGene (Rockville, MD) was used.

For Syk silencing, T cells were transfected with 15nM of either control siRNA or SYK-specific siRNA (Ambion, Grand Island, NY). Pre-designed and validated siRNA was purchased from Applied Biosystems (Grand Island, NY): SYK siRNA, sense, CGCUCUUAAAGAUGAGUUATT, and antisense, UAACUCAUCUUUAAGAGCGGG.

Following transfections, cells were rescued immediately in prewarmed RPMI medium supplemented with 10% fetal bovine serum and 1% penicillin and streptomycin in 24-well culture plates.

### RNA isolation and reverse transcription

Three million cells were lysed in RLT buffer and RNA was extracted using Qiagen (Valencia, CA) RNeasy extraction kit. A DNase-I treatment step (Qiagen) was added to the standard protocol to ensure exclusion of genomic DNA from the final product. OD_260/280_ measurements were used as a measure of quality of isolated RNA. T cell derived total RNA was reversely transcribed into cDNA using Promega (Madison, WI) reverse transcription system and a mixture of 1:10 oligo (dT)_20_ to random hexamer primers. Reverse transcription was performed in a conventional thermocycler.

### Real-time PCR

Quantitative real time polymerase chain reaction (rtPCR) was performed to measure gene expression levels using UPL probes (Universal Probe Library) from Roche (Indianapolis, IN). A reaction mixture of a total volume of 10µl was prepared in a final concentration of 200nM for each primer, 100nM for the probes, 1x LightCycler 480 Probes Master and cDNA. All reagents were obtained from Roche (Indianapolis, IN) apart from High Purity Salt Free (HPSF) primers obtained from Eurofins MWG Operon (Huntsville, AL). Reactions were prepared in 96 well plates and amplification was performed on a Roche LightCycler 480 PCR instrument (Roche, Indianapolis, IN). Detailed information on the primers and probes used is given in Table S1.

Crossing points (Ct) were calculated using the second derivative maximum method and expression levels were normalized against two reference genes (CD3ε and GAPDH). Ct values over 40 were excluded from the analysis.

### Immunofluorescent staining

Half a million cells from each blood donor were stained *ex vivo* for flow cytometry analysis. After harvesting, cells were incubated at room temperature for 30 min with cell surface fluorochrome-conjugated monoclonal antibodies. For intracellular staining, cells were then resuspended in 100 µl of Cytofix/Cytoperm solution (BD Biosciences, San Jose, CA) for 20 min at 4°C and washed twice in Perm/Wash solution (BD Biosciences, San Jose, CA). After 30min incubation with intracellular antibodies, cells were again washed and collected by centrifugation at 400xg for 5 minutes.

Expression of cell surface and intracellular markers was assessed on a BD Biosciences LSRII flow cytometer, and data were gated and displayed in Flowing Software 2.5 (Turku Centre for Biotechnology, Finland).

### Western blotting

Cells were first pelleted and then lysed in radioimmunoprecipitation assay (RIPA) buffer (Boston Bioproducts, Ashland, MA). Lysates were then resolved on 4–12% BisTris gels and transferred to polyvinylidene difluoride (PVDF) membrane. Membranes were then blocked with 4% nonfat milk in Tris-buffered saline with 0.05% Tween 20 (TBS-T) for 1h and incubated with primary antibody at room temperature for 1h. After washing three times with TBS-T membranes were then incubated with horseradish peroxidase-conjugated secondary antibody for 1h, washed three times and developed with ECL detection reagents (GE Healthcare, Piscataway, NJ). Bands were visualized in the Fujifilm LAS-4000 imager and densitometry performed in ImageJ software (National Institutes of Health).

### Analysis and statistical methods

Student’s t-test was used for statistical analysis. All plots were constructed in Prism 5 (GraphPad, La Jolla, CA).

## Results

### a) Modulation of Syk expression in SLE-patient and healthy blood-donor T cells

**Figure 1:**
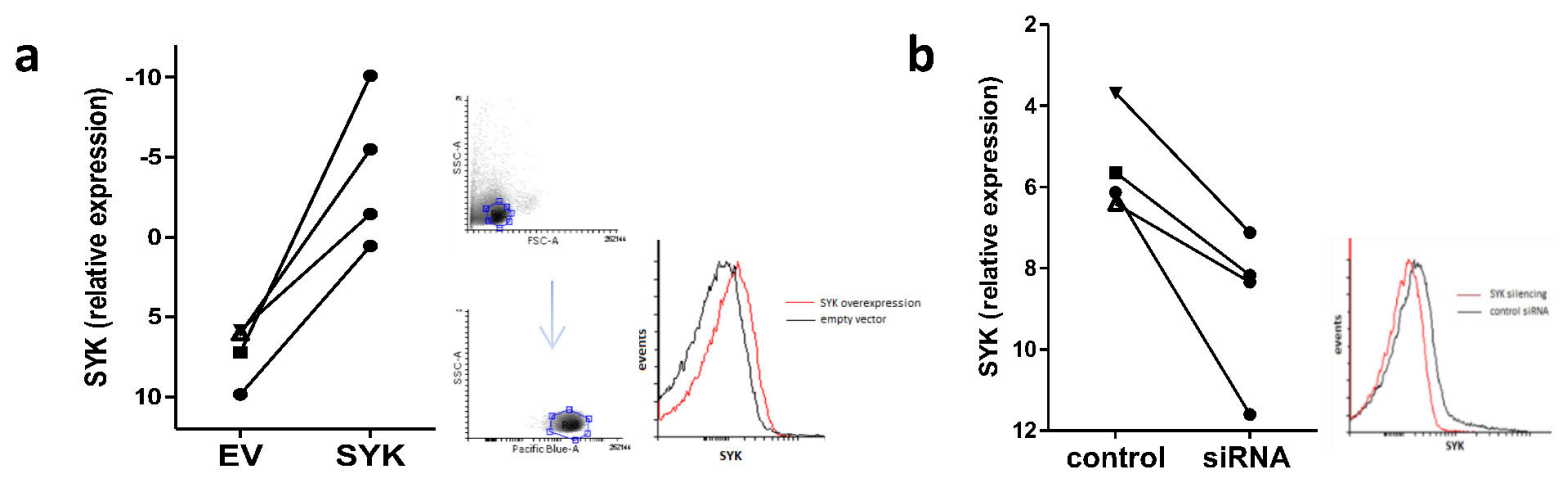
Induced Syk expression and Syk silencing in T cells. a) T cells extracted from healthy blood-donors were transfected with either a SYK (SYK) or an empty (EV) expression vector. Following 72h of incubation, cells were lysed, RNA extracted and SYK expression levels measured in real-time PCR. SYK overexpression led to a significant increase in its mRNA levels in all four experiments tested (left panel, normalized expression levels shown). To investigate whether this finding translates into protein expression levels as well, whole T cells were used to be analyzed in flow cytometry. Syk protein was found to significantly increase following SYK overexpression in all experiments tested (right panel, a representative plot is shown, plots gated on CD3+ T cells). b) T cells extracted from SLE patients were transfected with either a SYK-specific (siRNA) or a control (control) siRNA and expression levels of Syk were measured using real-time PCR and flow cytometry. The SYK silencing protocol led to a significant reduction in its expression at both the mRNA (left panel) and protein levels (right panel).

Although it is established that Syk is increased in SLE T cells it has not been previously shown whether increased Syk expression represents a primary or a secondary abnormality in these cells. To determine this we first upregulated Syk expression in healthy blood donor T cells using a SYK expression vector. Following transfection with the expression vector, cells were cultured for 72h and SYK messenger RNA (mRNA) was measured using quantitative PCR. Syk overexpression resulted in significant upregulation of Syk in all experiments performed. Average expression levels more than doubled in these cells in comparison to empty vector transfected (Figure 1a). To examine whether this finding translates into higher protein expression levels as well, Syk was measured by flow cytometry. Syk was significantly upregulated in those cells transfected with the SYK-expression vector in comparison to those transfected with the empty vector (Figure 1a).

To examine whether any of the abnormalities seen in SLE patients are corrected following downregulation of SYK expression we then used SLE patient T cells and silenced its expression using a SYK-specific small interfering RNA (siRNA). SYK mRNA levels were found to be significantly decreased in SYK-siRNA transfected T cells in comparison to control siRNA transfected ones (≥50%) (Figure 1b). Expression levels of Syk at the protein level were also found to significantly decrease following SYK silencing (Figure 1b). There was no correlation between SLEDAI score and fold Syk change following SYK silencing. These results demonstrate that SYK silencing was successful in downregulating intracellular Syk expression at the gene and protein levels.

### b) SYK affects expression levels of genes associated with SLE

**Figure 2:**
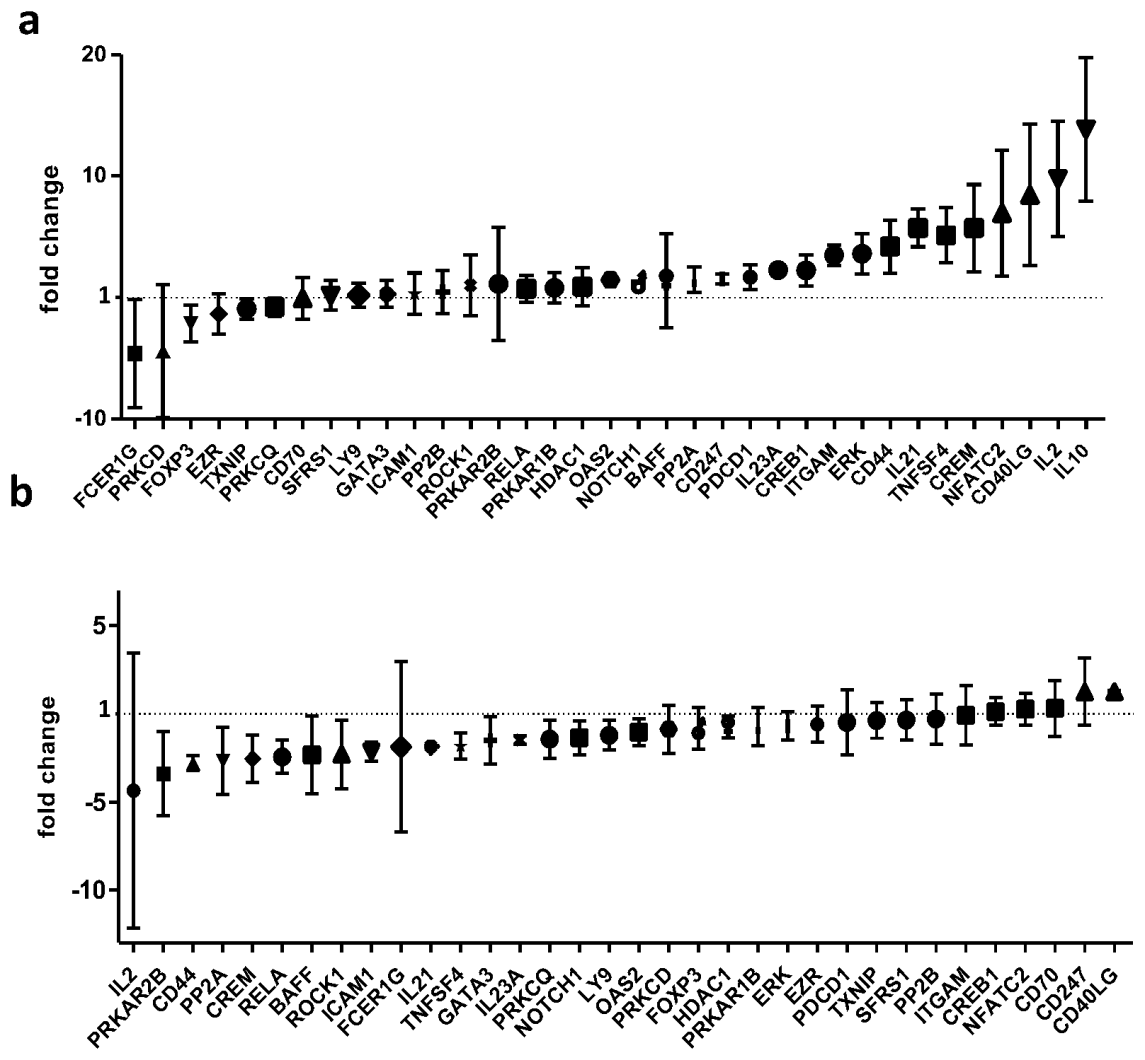
Intracellular levels of Syk affect the expression of genes associated with SLE immunopathogenesis. a) Healthy blood donor T cells were transfected with a SYK overexpression plasmid and the expression of an array of genes associated with SLE immunopathogenesis was measured using real-time PCR. A number of genes known to be overexpressed in SLE patients were found to be upregulated by SYK overexpression, like IL-21, CD44, OAS2 and PP2A (mean ±SEM fold expression changes between overexpression and empty vector transfected cells in four different experiments are shown). b) SLE patient T cells were transfected with either a SYK-specific siRNA or a control, non-silencing, siRNA. Silencing of SYK resulted in suppression of expression of a number of genes known to be aberrantly upregulated in SLE. Notably, expression levels of IL-21, CD44, OAS2 and PP2A were all found to decrease in SYK-knockdown T cells (mean ±SEM fold expression changes between silencing and control transfected T cells in four different experiments are shown).

To determine whether Syk regulates at the mRNA level the production of cell molecules that are known to be aberrantly expressed in SLE we used healthy blood donor T cells and SYK expression was artificially induced using a SYK-overexpression vector. A panel of 36 genes known to play an important role in aberrant SLE T cell function was chosen to be studied. We chose these genes because of their proven association with SLE pathophysiology and our own observations that this panel of genes can reliably differentiate between SLE patients and controls ( [11] and unpublished data). We indeed found that overexpression of SYK resulted in upregulation of several of these molecules (Figure 2a). Most notably, the expression of cytokine IL-21, cell surface molecule CD44, and intracellular molecules PP2A and OAS2 were found to substantially increase in cells overexpressing SYK (fold increase in SYK overexpressing cells over controls: IL-21, 5.7±1.5; CD44, 4.2±2.2; PP2A, 1.5±1; OAS2, 1.5±0.3; normalized against GAPDH and CD3ε).

Following the reverse approach, suppression of Syk expression in SLE T cells resulted in a substantial decrease in the expression of those genes (Figure 2b). Specifically, transcript levels of IL-21, PP2A, OAS2 and CD44 were all found to decrease following siRNA-mediated SYK knockdown (fold increase in SYK siRNA treated cells over controls: CD44, -2.8±0.4; PP2A, -2.7±1.9; IL-21, -1.9±0.2; OAS2, -1.1±0.8, normalized against GAPDH and CD3ε).

### c) Forced SYK expression in healthy blood donor T cells modulates expression levels of molecules associated with the pathogenesis of SLE

To verify that changes seen at the mRNA level translate into changes in protein expression, we measured the protein levels of CD44, IL-21, PP2A and OAS2 following overexpression and silencing of Syk. For CD44 in particular we measured splice variants v3 and v6, as both are associated with SLE [12].

**Figure 3:**
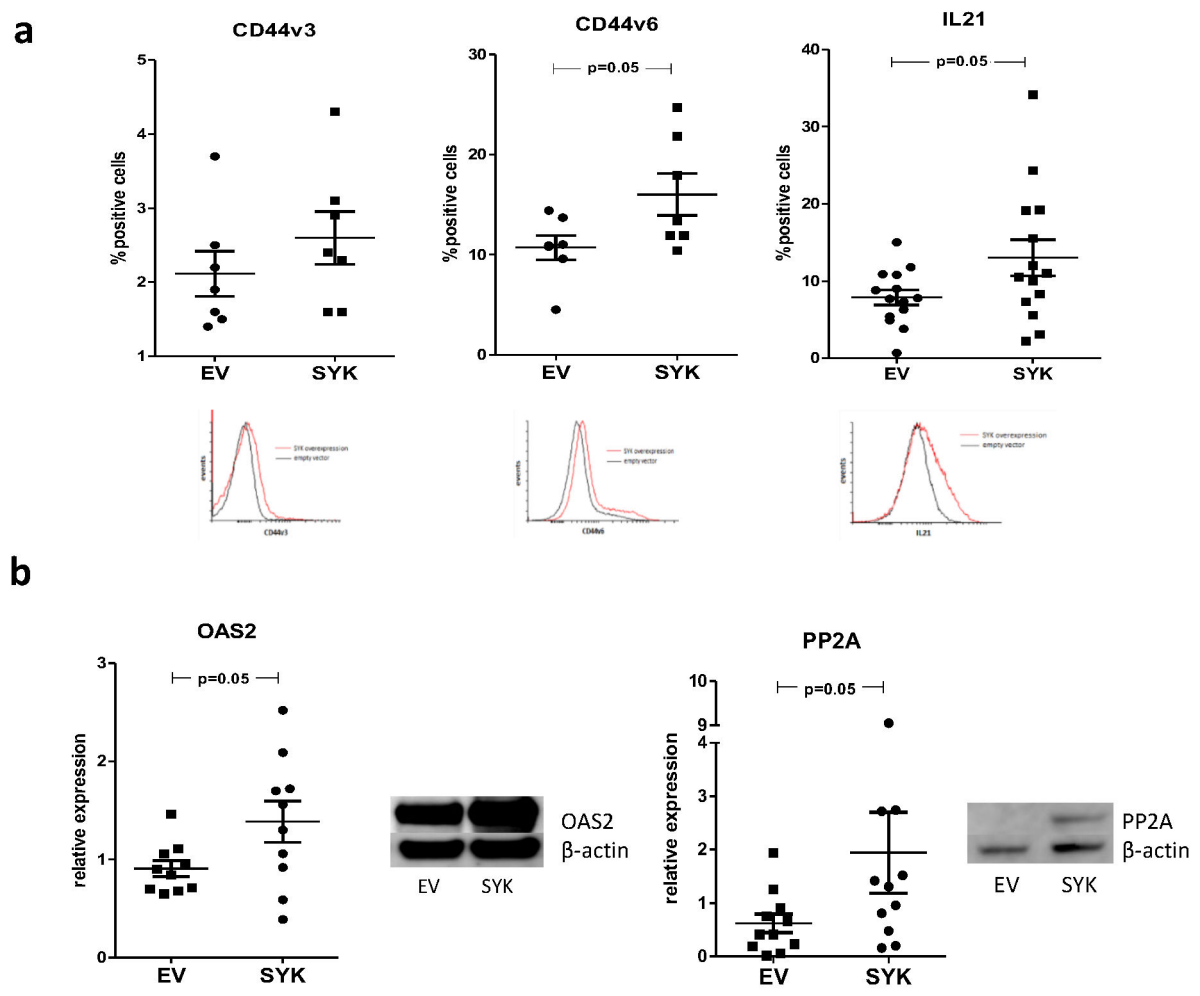
Induced Syk expression upregulates expression levels of SLE-associated molecules in healthy blood donor T cells. a) SYK was overexpressed in healthy blood donor T cells using a transient nucleofection protocol and expression levels of IL-21, CD44v3 and CD44v6 measured in flow cytometry. SYK overexpression resulted in the upregulation of the expression of all these molecules at the protein level. Changes in expression of variant v6 of CD44 molecule seemed to account for most of the changes seen at the mRNA level (bars show the mean ±SEM of SYK (SYK) vs. empty vector (EV) transfected T cells in ≥7 experiments). b) Following the same transfection protocol cells were lysed in RIPA buffer and subjected to western blot using antibodies against PP2a, OAS2 and β-actin. Protein expression levels of both OAS2 and PP2a increased significantly following SYK induced expression. Following measurement of band intensity the ratio of PP2a or OAS2 to β-actin was calculated in each experiment (a representative experiment and cumulative data (mean ± SEM) from 10 experiments are shown).

Overexpression of SYK in healthy blood donor T cells led to a significant upregulation in expression of IL-21 cytokine and CD44v6 receptor. A smaller increase in CD44v3 expression was also seen although overall levels of this variant were found to be quite low (Figure 3a, empty vector vs. SYK expressing vector transfected T cells, mean positive cells±SEM: IL-21, 7.9±1 vs. 13±2.3, p=0.05; CD44v3, 2.1±0.3 vs. 2.6±0.4, p = ns; CD44v6, 10.7±1.2 vs. 16±2.1, p=0.05).

To evaluate the effect of Syk forced expression on cytoplasmic molecules PP2A and OAS2, cells were lysed in RIPA buffer and subjected to Western blot. T cells transfected with the SYK cDNA construct displayed a significant increase in expression of both OAS2 and PP2A in comparison to empty vector transfected (Figure 3b, empty vector vs. SYK expressing vector transfected T cells, mean relative expression±SEM, OAS2/actin ratio: 0.91±0.1 vs. 1.39±0.2, p=0.05; PP2A/actin ratio: 0.49±0.1 vs. 1.23±0.3, p=0.05).

### d) Silencing of SYK in SLE patient T cells corrects disease-associated T cell abnormalities

**Figure 4:**
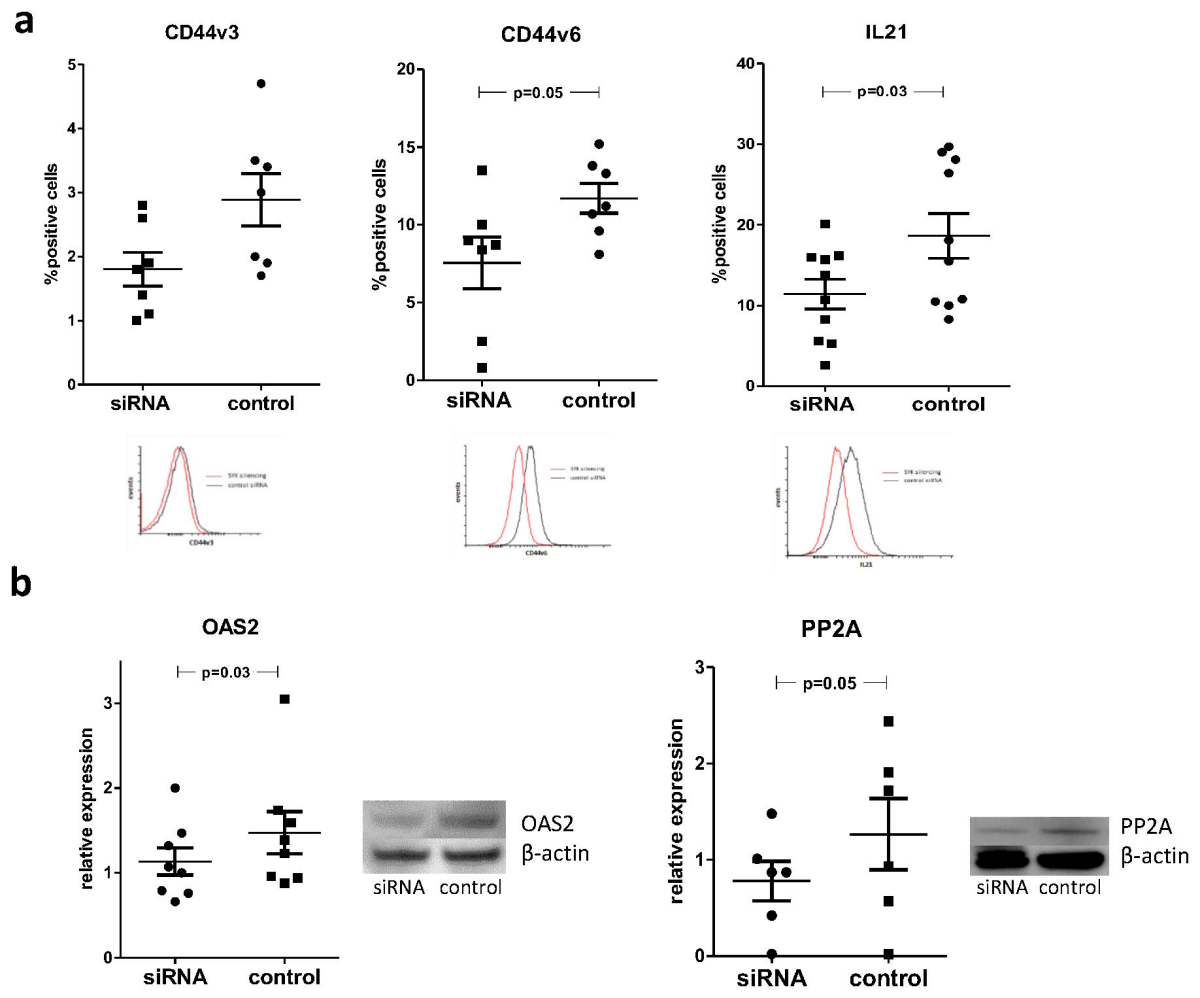
Silencing of SYK results in the suppression of aberrantly expressed molecules in SLE. a) SLE patient T cells were transfected with 15nM of either control or SYK-specific siRNA. Seventy-two hours following transfection cells were harvested and stained with antibodies against IL-21, CD44v3 and CD44v6. Silencing of SYK resulted in the suppression of expression of all the above molecules, particularly IL-21 and CD44v6. Bars show the mean ±SEM of control (control) vs. SYK-siRNA (siRNA) transfected T cells in ≥7 experiments and a representative experiment is shown. b) Protein expression levels of OAS2 and PP2a measured in western blot were found to decrease following silencing of SYK. Mean ±SEM of densitometry analyses of SYK-specific (siRNA) vs. control (control) siRNA transfected cells in ≥6 experiments are plotted. A representative experiment and cumulative data are shown.

We then measured protein expression of CD44v3, CD44v6, IL-21, PP2A and OAS2 in SYK-siRNA treated SLE T cells. Knockdown of SYK resulted in a significant decrease in CD44v6 and IL-21 expression. Again, the decrease in CD44v3 expression was found to be smaller and overall expression levels of this molecule quite low (Figure 4a, control vs. SYK siRNA transfected T cells, mean positive cells±SEM: IL-21, 18.6±2.4 vs. 11.4±1.6, p=0.03; CD44v3, 2.9±0.3 vs. 1.8±0.2, p = ns; CD44v6, 11.7±0.7 vs. 7.6±1.2, p=0.05).

T cells in which SYK had been silenced also displayed decreased expression of OAS2 and PP2A in comparison to control siRNA treated (Figure 4b, control vs. SYK siRNA transfected T cells, mean relative expression±SEM, OAS2/actin ratio: 1.47±0.2 vs. 1.13±0.1, p=0.03; PP2A/actin ratio: 1.26±0.3 vs. 0.78±0.2, p=0.05).

## Discussion

SLE T cells express high levels of Syk and preferentially transmit signals through FcRγ/Syk instead of the canonical CD3ζ/ Zap70 pathway. This rewiring of the TCR signaling complex is associated with profound transcriptional dysregulation of several key molecules in SLE T cells [13]. Specifically, SLE T cells display upon activation increased calcium flux, tyrosine phosphorylation and actin polymerization [14].

We have shown previously that Syk expression is controlled by the transcription factors c-Jun and Ets-2 and is transcriptionally upregulated in SLE T cells [15] primarily due to activated c-Jun. Syk inhibition using siRNA [15] or a small molecule R406/R788 [3] resulted in decrease in the calcium flux following SLE T cell activation, but had no effect on normal T cells. Moreover, Syk inhibition decreased the rapid actin polymerization of SLE T cells proving the importance of Syk in SLE T cell activation. The global importance of Syk in SLE was also shown in lupus prone mice, where treatment with R788 resulted in prevention of nephritis and dermatitis [16].

**Figure 5:**
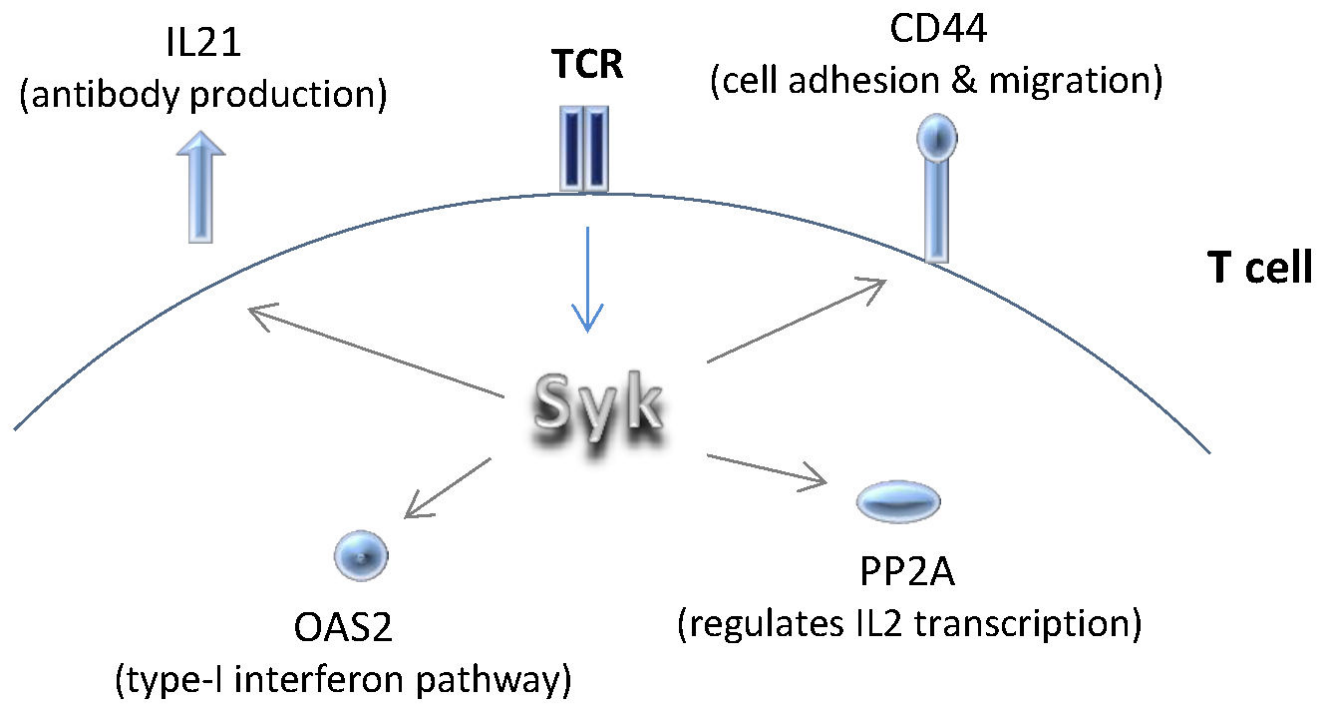
Schematic representation of the proposed role of Syk in SLE. Syk promotes the upregulation of a number of cytokines, receptors and enzymes that play a key role in SLE immunopathogenesis. It affects expression levels of CD44, primarily variant v6, involved in T cell migration; IL-21, involved in antibody production; OAS2, involved in type-I interferon responses; and PP2A, involved in the regulation of IL-2 production.

Given these findings we asked whether upregulation of Syk in normal T cells can re-create the phenotype of SLE T cells; and vice versa, whether downregulation of Syk can normalize the expression of key signaling molecules in SLE T cells. We chose to examine the expression of 39 signaling molecules that have been linked to SLE T cell phenotype. Of those molecules, four were most profoundly and consistently affected by Syk overexpression and downregulation (Figure 5). Specifically, overexpression of Syk resulted in upregulation of IL21, CD44, PP2A and OAS2. Silencing of SYK, on the other hand, resulted in downregulation of these molecules. Findings were consistent at both the mRNA and protein levels for all molecules tested. Out of the two CD44 receptor molecule variants most highly associated with SLE (v3 and v6) [12] v6 was found to be primarily affected by changes in Syk expression levels.

A number of previous studies have shown the importance of the above molecules in SLE. IL-21, has been found to play an important role in T cell-dependent B cell differentiation into plasma cells and the production of antibodies in SLE [17]. Hence Syk overexpressing SLE T cells can provide increased help to B cells to produce pathogenic autoantibodies, a key feature of the disease.

Expression of CD44, a cell-surface glycoprotein involved in cell-cell interactions and cell adhesion is increased in SLE T cells, allowing for increased adhesion and migration [18]. CD44 splice variants v3 and v6 in particular, are upregulated in SLE T cells and their expression correlates with disease activity [12]. T cells in kidneys of SLE patients have been found to express CD44 suggesting that these molecules may allow T cells to migrate abnormally into kidneys in them [19]. Therefore Syk not only controls actin polymerization upon SLE T cell activation, but also by enhancing CD44 expression may lead to faster adhesion and migration of T cells to tissues. Although both v3 and v6 variants are associated with SLE, we found that mainly variant v6 is regulated by Syk. This finding may demonstrate a variable role of Syk in the expression of these two splice variants and points towards an important role for CD44v6 in the pathogenesis of SLE.

PP2A (protein phosphatase 2), a serine/threonine phosphatase that regulates a number of cellular processes, plays an important role in SLE. SLE T cells express abnormally high levels of PP2A, which leads to decreased expression of interleukin-2 upon T cell activation [20]. As IL-2 is important for Treg function, increased expression of PP2A may be in part responsible for defective Treg function in SLE [21]. PP2A also regulates the expression of CD3ζ and FcRγ, leading to TCR rewiring [22] and further enhancing Syk recruitment to the TCR/FcRγ complex. Moreover, PP2A-transgenic mice display increased expression of IL-17 and increased susceptibility to immune-mediated glomerulonephritis [23]. Therefore, Syk by controlling at least in part the expression of PP2A, may regulate the composition of TCR and tilt the balance of T cell activation towards the pro-inflammatory Th17.

Finally, OAS2 (2'-5'-oligoadenylate synthetase) is an interferon-induced molecule that is involved in the innate immune response towards viral infection. OAS2 activates latent RNase L which is involved in the degradation of viral RNA. Along with several other interferon-inducible molecules, OAS2 has been found to be significantly upregulated in SLE [24]. These molecules represent the, so-called, ‘interferon-signature’ in SLE and are thought to be the result of high levels of type I Inteferons (IFNs) in these patients. Type I IFNs play an important role in the promotion of inflammatory responses; they prevent activated T-cell death and contribute to the generation of effector cells during viral infections [25]. They have also been found to play a role in the process of CD8 T cell-dependent generation of autoantigens [26].

Exactly how Syk affects the expression of these molecules remains to be determined. Previous studies suggest some possible links: Syk has been found to associate with adaptor molecules Vav1, phospholipase Cγ1 (PLCγ1) [27,28] and extracellular signal-regulated kinase (ERK) [29] in the process of regulating the production of cytokines and other molecules. Vav1 is a molecule known to activate NF-κB and, a member of the NF-κB family, c-Rel, has been found to activate transcription of IL-21 in T cells [30]. Finally, p38, a member of the mitogen-activated protein kinase (MAPK) pathway (which includes ERK) has been found to be involved in the expression of CD44 in monocytes [31].

In this study, we focused on determining the effect of Syk on resting T cells as we have previously shown the effect of Syk overexpression/knockdown on the activation of T cells [15]. Therefore, the effect of Syk on the expression of molecules that are produced by T cells upon activation (such as IL-2 or CD40 ligand) cannot be fully assessed.

In conclusion, our data show that overexpression of Syk in healthy T cells recapitulates at least part of the SLE T cell phenotype. Syk overexpressing T cells may provide more help to B cells through IL21, have enhanced migration to tissues by upregulating CD44 and produce proinflammatory rather than counterinflammatory cytokines. Inhibiting Syk in SLE T cells leads to the opposite effect, further underscoring Syk’s potential as a therapeutic target in SLE.

## Supporting Information

Table S1 **Assay characteristics of real-time PCR gene targets.**
(DOCX)

## References

[1] Liossis SN, Ding XZ, Dennis GJ, Tsokos GC (1998) Altered pattern of TCR/CD3-mediated protein-tyrosyl phosphorylation in T cells from patients with systemic lupus erythematosus. Deficient expression of the T cell receptor zeta chain. J Clin Invest 101: 1448-1457. doi:10.1172/JCI1457. PubMed: 9525988. 9525988 10.1172/JCI1457 PMC508723

[2] Tsokos GC (2011) Systemic lupus erythematosus. N Engl J Med 365: 2110-2121. doi:10.1056/NEJMra1100359. PubMed: 22129255. 22129255 10.1056/NEJMra1100359

[3] Krishnan S, Juang YT, Chowdhury B, Magilavy A, Fisher CU et al. (2008) Differential expression and molecular associations of Syk in systemic lupus erythematosus T cells. J Immunol 181: 8145-8152. PubMed: 19018007. 19018007 10.4049/jimmunol.181.11.8145 PMC2586973

[4] Kyttaris VC (2010) Systemic lupus erythematosus: from genes to organ damage. Methods Mol Biol 662: 265-283. doi:10.1007/978-1-60761-800-3_13. PubMed: 20824476. 20824476 10.1007/978-1-60761-800-3_13 PMC3153363

[5] Crispín JC, Kyttaris VC, Juang YT, Tsokos GC (2008) How signaling and gene transcription aberrations dictate the systemic lupus erythematosus T cell phenotype. Trends Immunol 29: 110-115. doi:10.1016/j.it.2007.12.003. PubMed: 18249583. 18249583 10.1016/j.it.2007.12.003

[6] Grammatikos AP, Tsokos GC (2012) Immunodeficiency and autoimmunity: lessons from systemic lupus erythematosus. Trends Mol Med 18: 101-108. doi:10.1016/j.molmed.2011.10.005. PubMed: 22177735. 22177735 10.1016/j.molmed.2011.10.005 PMC3278563

[7] Oliver JM, Burg DL, Wilson BS, McLaughlin JL, Geahlen RL (1994) Inhibition of mast cell Fc epsilon R1-mediated signaling and effector function by the Syk-selective inhibitor, piceatannol. J Biol Chem 269: 29697-29703. PubMed: 7961959. 7961959

[8] Nambiar MP, Fisher CU, Kumar A, Tsokos CG, Warke VG et al. (2003) Forced expression of the Fc receptor gamma-chain renders human T cells hyperresponsive to TCR/CD3 stimulation. J Immunol 170: 2871-2876. PubMed: 12626537. 12626537 10.4049/jimmunol.170.6.2871

[9] Hochberg MC (1997) Updating the American College of Rheumatology revised criteria for the classification of systemic lupus erythematosus. Arthritis Rheum 40: 1725. doi:10.1002/art.1780400929. PubMed: 9324032. 10.1002/art.1780400928 9324032

[10] Bombardier C, Gladman DD, Urowitz MB, Caron D, Chang CH (1992) Derivation of the SLEDAI. A disease activity index for lupus patients. The Committee on Prognosis Studies in SLE. Arthritis Rheum 35: 630-640. doi:10.1002/art.1780350606. 10.1002/art.1780350606 1599520

[11] Juang YT, Peoples C, Kafri R, Kyttaris VC, Sunahori K et al. (2011) A systemic lupus erythematosus gene expression array in disease diagnosis and classification: a preliminary report. Lupus 20: 243-249. doi:10.1177/0961203310383072. PubMed: 21138984. 21138984 10.1177/0961203310383072 PMC3880791

[12] Crispín JC, Keenan BT, Finnell MD, Bermas BL, Schur P et al. (2010) Expression of CD44 variant isoforms CD44v3 and CD44v6 is increased on T cells from patients with systemic lupus erythematosus and is correlated with disease activity. Arthritis Rheum 62: 1431-1437. doi:10.1002/art.27385. PubMed: 20213807. 20213807 10.1002/art.27385 PMC2879041

[13] Kyttaris VC, Tsokos GC (2011) Targeting lymphocyte signaling pathways as a therapeutic approach to systemic lupus erythematosus. Curr Opin Rheumatol 23: 449-453. doi:10.1097/BOR.0b013e328349a242. PubMed: 21720246. 21720246 10.1097/BOR.0b013e328349a242 PMC3158574

[14] Grammatikos AP, Tsokos CG (2011) T cell abnormalities in patients with SLE (systemic lupus erythematosus). World Hellenic Biomedical. News 2: 7-11.

[15] Ghosh D, Tsokos GC, Kyttaris VC (2012) c-Jun and Ets2 proteins regulate expression of spleen tyrosine kinase in T cells. J Biol Chem 287: 11833-11841. doi:10.1074/jbc.M111.333997. PubMed: 22354960. 22354960 10.1074/jbc.M111.333997 PMC3320931

[16] Deng GM, Liu L, Bahjat FR, Pine PR, Tsokos GC (2010) Suppression of skin and kidney disease by inhibition of spleen tyrosine kinase in lupus-prone mice. Arthritis Rheum 62: 2086-2092. PubMed: 20222110. 20222110 10.1002/art.27452 PMC2902591

[17] Sarra M, Monteleone G (2010) Interleukin-21: a new mediator of inflammation in systemic lupus erythematosus. J Biomed Biotechnol, 2010: 294582 PubMed: 20652041 10.1155/2010/294582 PMC2905909 20652041

[18] Li Y, Harada T, Juang YT, Kyttaris VC, Wang Y et al. (2007) Phosphorylated ERM is responsible for increased T cell polarization, adhesion, and migration in patients with systemic lupus erythematosus. J Immunol 178: 1938-1947. PubMed: 17237445. 17237445 10.4049/jimmunol.178.3.1938

[19] Cohen RA, Bayliss G, Crispin JC, Kane-Wanger GF, Van Beek CA et al. (2008) T cells and in situ cryoglobulin deposition in the pathogenesis of lupus nephritis. Clin Immunol 128: 1-7. doi:10.1016/j.clim.2008.04.004. PubMed: 18565470. 18565470 10.1016/j.clim.2008.04.004 PMC2497421

[20] Katsiari CG, Kyttaris VC, Juang YT, Tsokos GC (2005) Protein phosphatase 2A is a negative regulator of IL-2 production in patients with systemic lupus erythematosus. J Clin Invest 115: 3193-3204. doi:10.1172/JCI24895. PubMed: 16224536. 16224536 10.1172/JCI24895 PMC1253625

[21] Crispín JC, Tsokos GC (2009) Transcriptional regulation of IL-2 in health and autoimmunity. Autoimmun Rev 8: 190-195. doi:10.1016/j.autrev.2008.07.042. PubMed: 18723131. 18723131 10.1016/j.autrev.2008.07.042 PMC2655130

[22] Juang YT, Wang Y, Jiang G, Peng HB, Ergin S et al. (2008) PP2A dephosphorylates Elf-1 and determines the expression of CD3zeta and FcRgamma in human systemic lupus erythematosus T cells. J Immunol 181: 3658-3664. PubMed: 18714041. 18714041 10.4049/jimmunol.181.5.3658 PMC2662392

[23] Crispín JC, Apostolidis SA, Rosetti F, Keszei M, Wang N et al. (2012) Cutting edge: protein phosphatase 2A confers susceptibility to autoimmune disease through an IL-17-dependent mechanism. J Immunol 188: 3567-3571. doi:10.4049/jimmunol.1200143. PubMed: 22422882. 22422882 10.4049/jimmunol.1200143 PMC3324672

[24] Tang J, Gu Y, Zhang M, Ye S, Chen X et al. (2008) Increased expression of the type I interferon-inducible gene, lymphocyte antigen 6 complex locus E, in peripheral blood cells is predictive of lupus activity in a large cohort of Chinese lupus patients. Lupus 17: 805-813. doi:10.1177/0961203308089694. PubMed: 18755862. 18755862 10.1177/0961203308089694

[25] Pascual V, Farkas L, Banchereau J (2006) Systemic lupus erythematosus: all roads lead to type I interferons. Curr Opin Immunol 18: 676-682. doi:10.1016/j.coi.2006.09.014. PubMed: 17011763. 17011763 10.1016/j.coi.2006.09.014

[26] Blanco P, Pitard V, Viallard JF, Taupin JL, Pellegrin JL et al. (2005) Increase in activated CD8+ T lymphocytes expressing perforin and granzyme B correlates with disease activity in patients with systemic lupus erythematosus. Arthritis Rheum 52: 201-211. doi:10.1002/art.20745. PubMed: 15641052. 15641052 10.1002/art.20745

[27] Deckert M, Tartare-Deckert S, Couture C, Mustelin T, Altman A (1996) Functional and physical interactions of Syk family kinases with the Vav proto-oncogene product. Immunity 5: 591-604. doi:10.1016/S1074-7613(00)80273-3. PubMed: 8986718. 8986718 10.1016/s1074-7613(00)80273-3

[28] Xu S, Huo J, Lee KG, Kurosaki T, Lam KP (2009) Phospholipase Cgamma2 is critical for Dectin-1-mediated Ca2+ flux and cytokine production in dendritic cells. J Biol Chem 284: 7038-7046. PubMed: 19136564. 19136564 10.1074/jbc.M806650200 PMC2652331

[29] Dennehy KM, Willment JA, Williams DL, Brown GD (2009) Reciprocal regulation of IL-23 and IL-12 following co-activation of Dectin-1 and TLR signaling pathways. Eur J Immunol 39: 1379-1386. doi:10.1002/eji.200838543. PubMed: 19291703. 19291703 10.1002/eji.200838543 PMC2720084

[30] Chen G, Hardy K, Bunting K, Daley S, Ma L et al. (2010) Regulation of the IL-21 gene by the NF-kappaB transcription factor c-Rel. J Immunol 185: 2350-2359. doi:10.4049/jimmunol.1000317. PubMed: 20639489. 20639489 10.4049/jimmunol.1000317

[31] Gee K, Lim W, Ma W, Nandan D, Diaz-Mitoma F et al. (2002) Differential regulation of CD44 expression by lipopolysaccharide (LPS) and TNF-alpha in human monocytic cells: distinct involvement of c-Jun N-terminal kinase in LPS-induced CD44 expression. J Immunol 169: 5660-5672. PubMed: 12421945. 12421945 10.4049/jimmunol.169.10.5660

