# Supplementary material for: Spleen Tyrosine Kinase (Syk) Regulates Systemic Lupus Erythematosus (SLE) T Cell Signaling
Source: PLoS One. 2013 Aug 27;8(8):e74550. doi: 10.1371/journal.pone.0074550 (PMC3754955; doi:10.1371/journal.pone.0074550)
Supplement: Table S1 — Assay characteristics of real-time PCR gene targets. (DOCX) [file pone.0074550.s001.docx]

**Table S1.** Assay characteristics of real-time PCR gene targets.

| **Symbol** | **GenBank** | **UPL probe** | **Amp icon^*^** | **Intron span^*^** | **left primer** | **right primer** |
| --- | --- | --- | --- | --- | --- | --- |
| **BAFF** | NM_001145645 | 69 | 95 | 105 | gagaagctgccagcagga | ggagctggtggttcaaagatt |
| **CD247** | NM_000734.3 | 42 | 107 | 77636 | ggcacagttgccgattaca | aggcagtgagaatgacacca |
| **CD3E** | NM_000733 | 49 | 96 | 989 | caaggccaagcctgtgac | tcatagtctgggttgggaaca |
| **CD40LG** | NM_000074 | 2 | 125 | 3972 | tcatgaaaacgatacagagatgc | cttcgtctcctctttgtttaacatt |
| **CD44** | NM_000610 | 41 | 76 | 1860 | caagcaggaagaaggatggat | aacctgtgtttggatttgcag |
| **CD70** | NM_001252 | 42 | 83 | 705 | ggtgatctgcctcgtggt | ctacgtcccacccaagtgac |
| **CREB1** | NM_004379 | 9 | 130 | 4950 | tgccaaggattgaagaagaga | attgctcctccctgggtaat |
| **CREMa** | NM_183060 | 9 | 124 | 27618 | actagcacggggcaatacac | gctggggactgtgcaaac |
| **ERK** | NM_002745.4 | 20 | 81 | 4951 | caaagaactaatttttgaagagactgc | tcctctgagcccttgtcct |
| **EZR** | NM_003379 | 9 | 123 | 1811 | gttgaagagtggcagcacag | gctcaccggctcgtacac |
| **FCER1G** | NM_004106 | 81 | 72 | 2612 | tgctcttactccttttggttga | tggcatccaggatatagcaga |
| **FOXp3** | NM_014009 | 20 | 85 | 1361 | gagaagctgagtgccatgc | agcccttgtcggatgatg |
| **GAPDH** | NM_002046 | 60 | 66 | 1631 | agccacatcgctcagacac | gcccaatacgaccaaatcc |
| **GATA3** | NM_002051.2 | 71 | 67 | 5333 | ctcattaagcccaagcgaag | tctgacagttcgcacaggac |
| **HDAC1** | NM_004964 | 81 | 90 | 564 | cggtgctggacatatgagac | tggtccaaagtattcaaagtagtca |
| **ICAM1** | NM_000201 | 71 | 90 | 8451 | ccttcctcaccgtgtactgg | agcgtagggtaaggttcttgc |
| **IL10** | NM_000572 | 30 | 60 | 1013 | ctgggggagaacctgaaga | gggaagaaatcgatgacagc |
| **IL2** | NM_000586 | 69 | 113 | 2287 | aagttttacatgcccaagaagg | aagtgaaagtttttgctttgagcta |
| **IL21** | NM_021803 | 7 | 68 | 2753 | aggaaaccaccttccacaaa | gaatcacatgaagggcatgtt |
| **IL23A** | NM_016584 | 47 | 102 | 164 | agggagatgaagagactacaaatga | tcctttgcaagcagaactga |
| **ITGAM** | NM_001145808 | 9 | 70 | 1300 | ggcatccgcaaagtggta | ggatcttaaaggcattctttcg |
| **LY9** | NM_001033667 | 4 | 73 | 3500 | ttctcttctgttctacagacctctctc | gggctgagtcctttccaga |
| **NFATC2** | NM_012340 | 8 | 144 | 41122 | ccagtcagtcaggctcttacg | ccatgtagccatggagctg |
| **NOTCH1** | NM_017617.3 | 90 | 90 | 968 | ctgcctgtctgaggtcaatg | tcacagtcgcacttgtaccc |
| **OAS2** | NM_016817.2 | 4 | 74 | 1187 | tcctcaacgaaagtgtcagc | tgtggagccagaactcagc |
| **PDCD1** | NM_005018 | 7 | 101 | 5780 | cggccaggatggttcttag | gtgaaggtggcgttgtcc |
| **PPP2CA** | NM_002715 | 47 | 108 | 770 | tcttcctctcactgccttgg | tcttgtaggcgatcaagtgc |
| **PPP2CB** | NM_004156 | 16 | 74 | 3297 | ccacttacagctttagtagatggaca | tccagtgtgtctatggatggag |
| **PRKAR1B** | NM_002735 | 24 | 129 | 9885 | atcaaggggaagtggatgtg | ggtccgtcttggctttcac |
| **PRKAR2B** | NM_002736 | 2 | 79 | 4458 | ctgctacctctcctggtgct | tttttggcattgtttttcaca |
| **PRKCD** | NM_006254 | 7 | 88 | 1290 | ggtgaagcagggattaaagtgt | gttgatgccgcagaggtt |
| **PRKCQ** | NM_006257 | 4 | 78 | 3497 | agctctactcgctggctga | ggccttgaggtttcagctct |
| **PTGS2** | NM_000963 | 69 | 109 | 426 | tgggaagccttctctaacctc | ttgaatcaggaagctgctttt |
| **RELA** | NM_021975 | 47 | 96 | 1468 | cgggatggcttctatgagg | ctccaggtcccgcttctt |
| **ROCK1** | NM_005406 | 9 | 88 | 3622 | cagtccttgggttgttcagc | aagatctccaccaggcatgtat |
| **SYK** | NM_003177 | 40 | 104 | 16610 | aaagacaaatggaaagttcctga | ctttgtcgatgcgatagtgc |
| **SFRS1** | NM_006924.4 | 16 | 63 | 367 | gcggtctgaaaacagagtgg | tttaaatcctgccaacttcca |
| **TNFSF4** | NM_003326.3 | 49 | 126 | 18447 | gccaagattcgagaggaaca | cgaggataccgatgtgatacc |
| **TXNIP** | NM_006472.3 | 42 | 114 | 111 | gcagatcaggtctaagcagca | ccatatagcagggaggagctt |

^*^ length in base pairs
